# Profiling and Identification of Small rDNA-Derived RNAs and Their Potential Biological Functions

**DOI:** 10.1371/journal.pone.0056842

**Published:** 2013-02-13

**Authors:** Haibin Wei, Ben Zhou, Fang Zhang, Yanyang Tu, Yanan Hu, Baoguo Zhang, Qiwei Zhai

**Affiliations:** Key Laboratory of Nutrition and Metabolism, Institute for Nutritional Sciences, Shanghai Institutes for Biological Sciences, Chinese Academy of Sciences, Shanghai, People’s Republic of China; Wageningen University, The Netherlands

## Abstract

Small non-coding RNAs constitute a large family of regulatory molecules with diverse functions. Notably, some small non-coding RNAs matched to rDNA have been identified as qiRNAs and small guide RNAs involved in various biological processes. However, a large number of small rDNA-derived RNAs (srRNAs) are usually neglected and yet to be investigated. We systematically investigated srRNAs using small RNA datasets generated by high-throughput sequencing, and found srRNAs are mainly mapped to rRNA coding regions in sense direction. The datasets from immunoprecipitation and high-throughput sequencing demonstrate that srRNAs are co-immunoprecipitated with Argonaute (AGO) proteins. Furthermore, the srRNA expression profile in mouse liver is affected by diabetes. Overexpression or inhibition of srRNAs in cultured cells shows that srRNAs are involved in various signaling pathways. This study presents a global view of srRNAs in total small RNA and AGO protein complex from different species, and demonstrates that srRNAs are correlated with diabetes, and involved in some biological processes. These findings provide new insights into srRNAs and their functions in various physiological and pathological processes.

## Introduction

Since the discovery of the first small silencing RNA in 1993 [1], a remarkable number of small RNA classes have been discovered, including microRNAs (miRNAs) [2], small interfering RNAs (siRNAs) [3], and Piwi-associated small RNAs (piRNAs) [4], [5], which have important roles in various biological processes. The discovery of new classes of small RNAs and new members of existing classes substantially expands our knowledge to small RNAs. For instance, a class of small RNAs originated from small nucleolar RNAs (snoRNAs) have been identified to function like miRNAs [6]. Notably, a new type of siRNA, known as qiRNAs (QDE-2-interacting small RNAs), originates mostly from the rDNA locus and has roles in DNA damage response in the filamentous fungus *Neurospora crassa* [7]. Moreover, other novel classes of small RNA are being revealed, including *cis*-acting siRNA (casiRNA), *trans*-acting siRNA (tasiRNA), natural antisense transcript siRNA (natsiRNA), exogenous siRNA (exo-siRNA) and endogenous siRNA (endo-siRNA) [8]. Besides, there are also a number of small RNA classes, such as heterochromatic siRNA (hc-siRNA) [9], stem bulge RNA (sbRNA) [10], vault RNA (vtRNA) [11], small scan RNA (scnRNA) [12], Y RNA [13] and DSB-induced small RNA (diRNA) [14]. However, many small RNAs obtained from high-throughput sequencing are not in the known classes [15], [16], [17], suggesting there are still some unknown classes of small RNAs.

Eukaryotic rRNAs are usually synthesized as long primary transcripts containing several different rRNAs separated by spacer regions, and mature rRNA molecules participate in the assembly of ribosomal subunits [18], [19]. Mammalian rDNA genes coding rRNAs are generally comprised of several hundreds of transcription units organized in tandem repeats and clustered on a number of chromosomal loci [20], [21], [22]. For example, in humans, there are approximately 300–400 rDNA repeats in five clusters on chromosomes 13, 14, 15, 21 and 22 [23]. Interestingly, rRNA is extremely abundant and makes up about 80% of the total RNA in eukaryotic cytoplasm [24], whereas full-length rDNA sequences are not included in human and mouse genome assembly [25]. Previous high-throughput sequencing studies tended to discard short RNA sequences mapping to rRNA as degradation products from further analysis [15], [16], [17], [26]. However, qiRNAs induced by DNA damage are mainly originated from rDNA locus [7], and a few of 28S rRNA fragments were very closely related to piRNAs, which form a hook structure and potentially work as small guide RNA [27]. It has been suggested that small rDNA-derived RNAs (srRNAs) can be produced as diRNAs [14]. These reports suggest that a lot of srRNAs are neglected and need further investigation.

To systematically investigate srRNAs, we analyzed some small RNA high-throughput sequencing datasets obtained from GEO at NCBI, and found srRNAs are mainly distributed in rDNA regions coding rRNAs. Moreover, srRNAs are existed in Argonaute (AGO) protein complex. In addition, some srRNAs are correlated with diabetes, and involved in regulation of metabolism and other biological processes. Our results provide a global view of srRNAs and their potential roles in physiological and pathological processes.

## Results

### Identification and characterization of mouse and human srRNAs from high-throughput sequencing datasets

**Figure 1:**
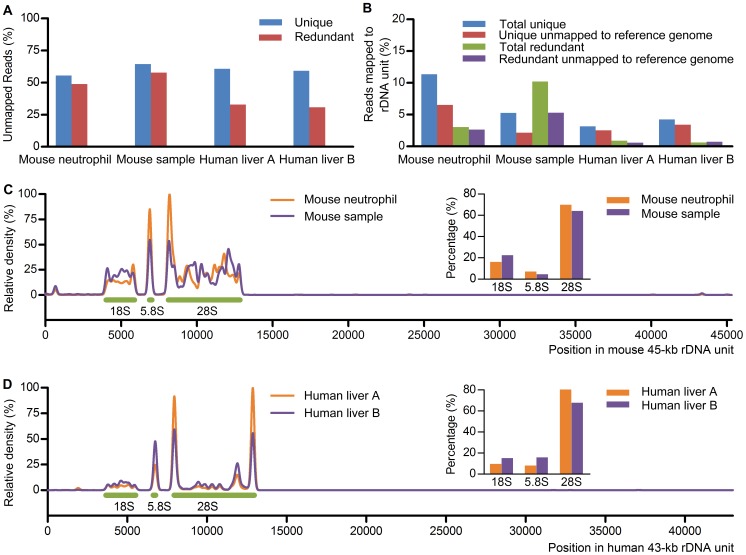
Identification of mouse and human small RNAs unmapped to reference genome but mapped to rDNA. (A) Percentage of unique and redundant small RNAs unmapped to reference genome. Mouse neutrophil (GSM304914), young mouse sample (GPL7059) and human liver (GSM531975 and GSM531976) small RNA data obtained from high-throughput sequencing were randomly collected from GEO. (B) Percentage of small RNAs mapped to the 45-kb mouse or 43-kb human rDNA unit. The blue and green histograms present the percentage of unique and redundant reads mapped to rDNA in total reads. The red and purple histograms show the percentage of unique and redundant reads mapped to rDNA among those unmapped to reference genome. (C and D) A continuous tag sequence density estimation by F-Seq showed that srRNAs from mouse (C) and human (D) were mainly enriched in the regions coding 18S, 5.8S and 28S rRNA.

To systematically investigate mouse and human srRNAs from different samples, we randomly selected 4 small RNA high-throughput sequencing datasets from GEO, 2 for mouse and 2 for human. As shown in Figure 1A, about 55–64% unique reads were unmapped to corresponding reference genome, and about 31–58% redundant reads were unmapped to corresponding reference genome. Although the complete sequences of the 45-kb mouse rDNA gene (GenBank: BK000964) and the 43-kb human rDNA gene (GenBank: U13369) have been reported [28], [29], rDNA is not included in genome assemblies. Interestingly, about 2.2–6.5% unique reads and 0.6–5.3% redundant reads among those unmapped to reference genome were mapped to rDNA unit (Figure 1B), and there were more unique and redundant reads in total reads are mapped to rDNA unit (Figure 1B). The Venn diagrams showed the overlap between the reads mapped to rDNA unit and those mapped to reference genome (Figure S1). The srRNAs in the four datasets were listed in Table S1,S2,S3,S4, and the srRNAs were named according to their position in rDNA and length.

In these srRNAs, averagely more than 99.8% were in sense orientation. About 16.1–22.4%, 4.5–7.0% and 64.0–70.0% srRNAs were distributed in mouse rDNA regions coding 18S, 5.8S and 28S rRNA respectively (Figure 1C), and about 9.6–15.1%, 8.1–15.8% and 67.6–80.4% srRNAs were distributed in human rDNA regions coding 18S, 5.8S and 28S rRNA respectively (Figure 1D). To intuitively display the distribution of srRNA in rDNA, we applied F-Seq software package to generate a continuous tag sequence density estimation. As shown in Figure 1C and 1D, srRNAs were mainly distributed in the rRNA coding regions, and srRNAs from the same species had similar distribution pattern in rDNA unit. This distribution pattern in rDNA unit was also observed in the other mouse and human small RNA datasets (Figure S2).

**Figure 2:**
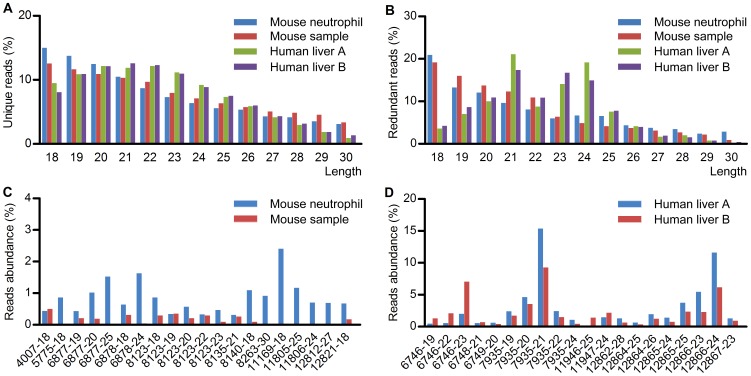
Size and frequency distribution of the srRNAs as well as the top 20 individual srRNAs. (A) A histogram of size distribution of 18–30 nt unique srRNAs in the indicated mouse and human samples. (B) Cumulative length distribution of srRNAs in the indicated samples. (C) The top 20 abundantly expressed srRNAs in the indicated mouse samples. (D) The top 20 expressed srRNAs in the indicated human samples.

The size distribution of 18–30 nt srRNAs was mainly enriched in 18–25 nt for both unique reads and redundant reads (Figure 2A and 2B). Generally, the size distribution was similar between the two human or mouse samples. The two human srRNA samples had the highest abundance at the size of 21 nt, which was different from the two mouse srRNA samples. The highest abundance of human srRNAs at the size of 21 nt suggests that a large number of srRNAs are not likely generated from random degradation.

The top 20 abundant srRNAs in the mouse or human samples according to their average abundance were shown in Figure 2C and 2D, and the details about the top 20 abundant srRNAs were shown in Table S5 and S6. The obvious different abundance of srRNAs further suggests that a large number of srRNAs are not likely generated from random degradation.

### srRNAs in AGO protein complex

**Figure 3:**
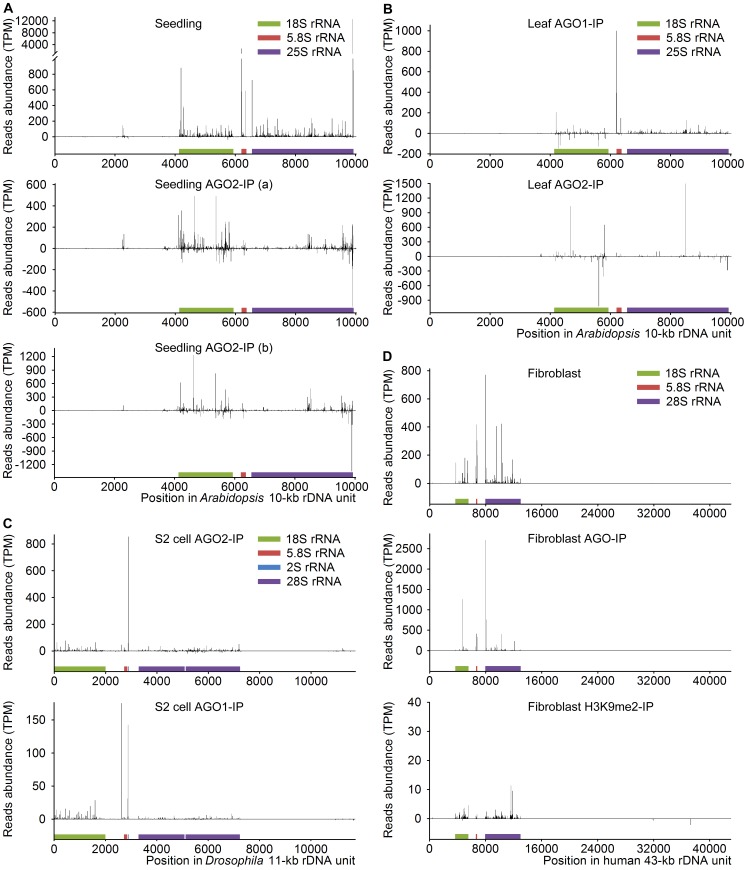
srRNAs co-immunoprecipitated with AGO proteins. (A) *Arabidopsis* srRNAs co-immunoprecipitated with AGO2. Total small RNA (GSM889256) or small RNA in the immunoprecipitated AGO2 complex (GSM889279) from Col-0 13-day-old seedlings were aligned to *Arabidopsis* rDNA unit. GSM889279a (a) and GSM889279b (b) are two biological replicates, indicating the similar distribution pattern for srRNAs co-immunoprecipitated with AGO2 compared with the different pattern of srRNAs from total seedling small RNA. (B) *Arabidopsis* srRNAs co-immunoprecipitated with AGO1 and AGO2 displayed a markedly different pattern. Small RNAs in the immunoprecipitated AGO1 complex (GSM642335) or AGO2 complex (GSM642337) from Col-0 4-week-old leaf tissue were aligned to *Arabidopsis* rDNA unit. (C) *Drosophila* srRNAs co-immunoprecipitated with AGO1 and AGO2 also displayed a markedly different pattern. Small RNAs in the immunoprecipitated AGO1 complex (GSM280088) or AGO2 complex (GSM280087) from S2 cells were aligned to *Drosophila* rDNA unit. (D) Human srRNAs were specifically co-immunoprecipitated with AGO proteins. Total small RNA (GSM850202) and small RNA co-immnoprecipitated with antibody against AGO proteins (GSM850203) or H3K9me2 (GSM850204) from human senescent fibroblast WI-38 were aligned to human rDNA unit. Of note, srRNAs immnoprecipitated with antibody against H3K9me2 showed very low abundance and nearly random distribution pattern, suggesting these srRNAs are from non-specific binding.

It was reported that AGO proteins can bind small non-coding RNAs and control protein synthesis, affect messenger RNA stability and even participate in the production of piRNA [30]. To investigate whether AGO proteins also bind srRNAs like many other small non-coding RNAs, high-throughput sequencing datasets with AGO protein precipitation were collected from various species, including *Arabidopsis* [14], [31], *Drosophila* [32] and human [33]. Analysis of *Arabidopsis* small RNAs co-immunoprecipitated with AGO2 from seedlings showed that srRNAs in AGO2 complex had a similar distribution pattern in two biological replicates, but had a markedly different pattern compared with srRNAs from total small RNA (Figure 3A). Moreover, *Arabidopsis* srRNAs in 4-week-old leaf tissue were co-immunoprecipitated with AGO1, but displayed a markedly different pattern compared with srRNAs co-immunoprecipitated with AGO2 in the same biological sample (Figure 3B). *Drosophila* srRNAs co-immunoprecipitated with AGO1 and AGO2 also showed a markedly different pattern (Figure 3C). Furthermore, Human srRNAs were also co-immunoprecipitated with AGO proteins, and showed an obvious different pattern compared to srRNAs in total small RNA (Figure 3D). Notably, srRNAs immnoprecipitated with antibody against H3K9me2 were mainly from non-specific binding, as evidenced by very low abundance and nearly random distribution pattern (Figure 3D). The reads and percentages of unique and total srRNA in the above samples were summarized in Table S7, and the srRNAs co-immunoprecipitated with AGO proteins were listed in Table S8. These data show AGO proteins can bind srRNAs, implicating many srRNAs function in AGO protein complex.

**Figure 4:**
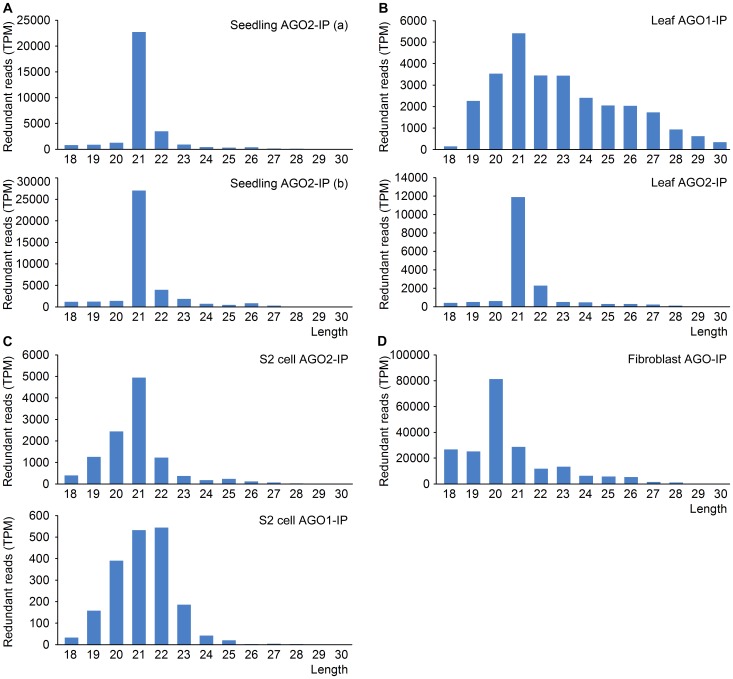
The length distribution of srRNAs in AGO protein complex. (A) The length distribution of *Arabidopsis* seedling srRNAs co-immunoprecipitated with AGO2 (GSM889279). GSM889279a (a) and GSM889279b (b) are two biological replicates. (B) The length distribution of *Arabidopsis* leaf srRNAs co-immunoprecipitated with AGO1 (GSM642335) and AGO2 (GSM642337) displayed a markedly different pattern. (C) The length distribution of *Drosophila* S2 cell srRNAs co-immunoprecipitated with AGO1 (GSM280088) and AGO2 (GSM280087) also displayed a markedly different pattern. (D) The length distribution of human fibroblast srRNAs co-immunoprecipitated with AGO proteins (GSM850203).

Generally, the size distribution of the srRNAs in AGO protein complex was mainly enriched in 20–22 nt (Figure 4), which is similar as miRNAs with an average length of 22 nt [34]. The length distribution of *Arabidopsis* seedling srRNAs in AGO2 complex from two biological replicates was almost the same with a peak at 21 nt (Figure 4A), which was obviously different from the length distribution of seedling total srRNAs (Figure S3A). Interestingly, the length distribution of *Arabidopsis* leaf srRNAs obtained from another dataset still almost had the same pattern as the seedling srRNAs (Figure 4B, lower panel). However, the length distribution of *Arabidopsis* srRNAs co-immunoprecipitated with AGO1 displayed an obviously different pattern (Figure 4B, upper panel). The length distribution of *Drosophila* srRNAs co-immunoprecipitated with AGO1 and AGO2 also showed a different pattern (Figure 4C). The srRNAs in human fibroblast AGO protein complex was mainly enriched in 20 nt (Figure 4D), which was different from the length distribution of the total human fibroblast srRNAs and human liver srRNAs (Figure S3B). The different length distribution of srRNAs in different AGO protein complexes provides further evidence that srRNAs in AGO protein complex are not mainly from random degradation.

### Expression profile of srRNA in mouse liver is correlated with diabetes

**Figure 5:**
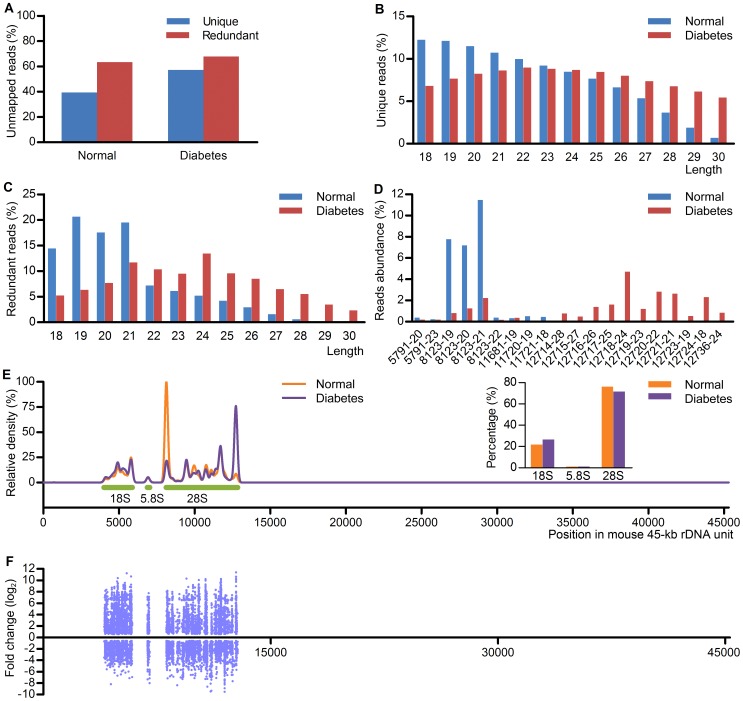
Hepatic srRNA expression profile is markedly changed in diabetic mice. (A) Percentage of unique and redundant hepatic small RNAs unmapped to reference genome from 9-week-old normal and diabetic mice. (B) Size distribution of 18–30 nt unique hepatic srRNAs in normal and diabetic mice. (C) Length distribution of the redundant srRNAs. (D) The top 20 abundantly expressed hepatic srRNAs in normal and diabetic mice. (E) Density estimation by F-Seq showed that hepatic srRNAs from normal and diabetic mice were mainly enriched in the regions coding 18S, 5.8S and 28S rRNA. (F) The distribution of differentially expressed hepatic srRNAs in normal and diabetic mice with FDR < 0.01 and fold change > 1.5 in the 45-kb mouse rDNA unit.

Many small RNAs are correlated with disease [35]. We hypothesized that srRNAs are also correlated with disease. To investigate the relationship between srRNAs and disease, we measured the small RNA expression profile of wild-type and diabetic mouse liver by high-throughput sequencing. The high-throughput sequencing data showed that there were about 39.4–57.2% unique reads and 63.3–67.7% redundant reads unmapped to mouse reference genome (Figure 5A). The srRNAs in the two datasets were listed in Table S9 and S10.

Generally, the size distribution of 18–30 nt srRNAs from normal and diabetic mouse liver was different for both unique and redundant reads (Figure 5B and 5C). Unique and redundant reads were peaked at size of 18 and 19 nt respectively for normal sample and 22 and 24 nt respectively for diabetic sample (Figure 5B and 5C). The top 20 abundant srRNAs in the two samples according to their average abundance were shown in Figure 5D, and the details about the top 20 abundant srRNAs were shown in Table S11.

About 21.9–26.6%, 1.0–1.1% and 71.5–76.1% srRNAs were distributed in mouse rDNA regions coding 18S, 5.8S and 28S rRNA respectively (Figure 5E). A continuous tag sequence density estimation by F-Seq was shown in Figure 5E, and srRNAs from normal and diabetic mouse liver were mainly distributed in the rDNA region coding rRNAs, and had similar peak positions.

The differentially expressed srRNAs (FDR < 0.01, fold change > 1.5) between normal and diabetic mouse liver were shown in Figure 5F. Totally 7664 srRNAs were differentially expressed, of which 4448 srRNAs were upregulated while 3216 srRNAs were downregulated (Table S12). These data demonstrate that the expression of srRNAs is correlated with diabetes, suggesting that srRNAs are involved in the pathogenesis of diabetes.

### The potential biological functions of srRNAs

**Figure 6:**
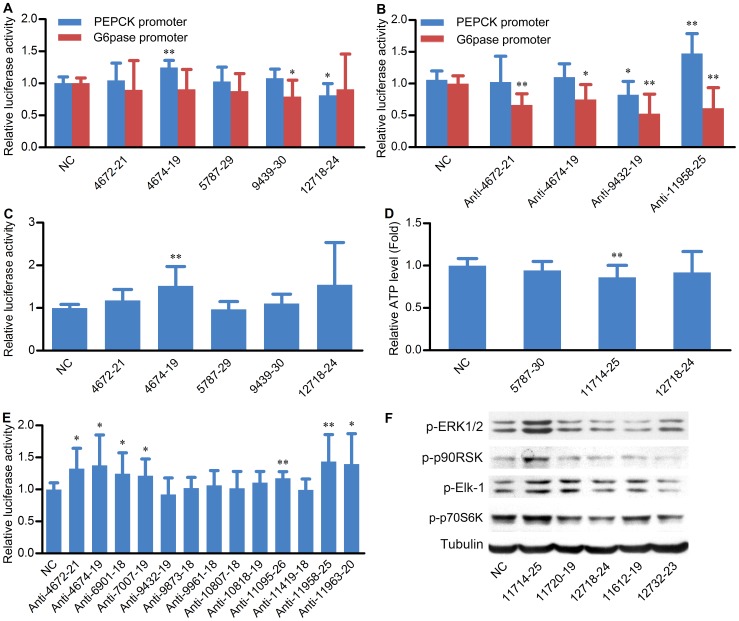
The various biological functions of srRNAs. (A) The effect of the selected srRNA mimics on PEPCK and G6Pase promoter activity. After transfection with the indicated srRNA and luciferase reporter for 72 h, Hepa 1–6 cells were harvested for luciferase assay. In this and all other figures, error bars represent SD. (B) The effect of the selected srRNA inhibitors on PEPCK and G6Pase promoter activity. After transfection with the indicated srRNA mimics and luciferase reporter for 72 h, Hepa 1–6 cells were harvested for luciferase assay. (C) The effect of the selected srRNA inhibitors on PPARγ promoter activity. After transfection with the indicated srRNA inhibitor and PPARγ promoter luciferase reporter for 72 h, Hepa 1–6 cells were harvested for luciferase assay. (D) The effect of the selected srRNA mimics on intracellular ATP levels. After transfection with the indicated srRNA mimics for 72 h, Hepa 1–6 cells were harvested for the measurement of ATP. (E) The effect of the selected srRNA inhibitors on PUMA promoter activity. After transfection with the indicated srRNA inhibitor and PUMA promoter luciferase reporter for 72 h, NIH/3T3 cells were harvested for luciferase assay. (F) The effect of the selected srRNA mimics on ERK pathway including phosohorylation of Erk1/2, p90RSK, Elk-1 and p70S6K. After transfection with the indicated srRNA mimic for 72 h, Hepa 1–6 cells were harvested for western blot. Tubulin was measured as internal control. *p < 0.05, **p < 0.01 versus negative control (NC).

Since the expression of srRNA is correlated with diabetes, we speculated that srRNA should be involved in some biological functions, such as glucose metabolism. PEPCK and G6pase are key gluconeogenic enzyme genes, and play important roles in glucose homeostasis [36]. To investigate the effect of some srRNAs correlated with diabetes on transcription of PEPCK and G6Pase, we transfected PEPCK and G6pase promoter luciferase reporters with the selected srRNA mimics or their inhibitors into mouse hepatoma cell line Hepa 1–6 cells. As shown in Figure 6A, overexpression of srRNA-12718-24 decreased the luciferase activity of PEPCK promoter reporter, while srRNA-4674-19 increased the luciferase activity of PEPCK promoter reporter. In addition, srRNA-9439-30 downregulated the luciferase activity of G6pase promoter reporter (Figure 6A). Interestingly, inhibition of srRNA-4672-21, srRNA-4674-19, srRNA-9432-19 or srRNA-11958-25 inhibited the G6Pase promoter reporter, and inhibition of srRNA-9432-19 also decreased the luciferase activity of PEPCK promoter reporter significantly (Figure 6B). Nevertheless, inhibition of srRNA-11958-25 increased the luciferase activity of PEPCK promoter reporter (Figure 6B). Since the sequences of srRNAs match with mature rRNAs, we detected whether srRNA inhibitors could downregulate mature rRNA levels. We found that there was no obvious difference of rRNA levels between the cells transfected with srRNA inhibitors matched with 18S and 28S rRNAs respectively or their controls, suggesting srRNA inhibitors have no significant effect on mature rRNA levels (Figure S4). PPARγ has been reported to regulate lipid and glucose metabolism [37], [38]. To investigate the effect of some srRNAs correlated with diabetes on PPARγ, we transfected PPARγ promoter luciferase reporter with the indicated srRNA inhibitors into Hepa 1–6 cells. As shown in Figure 6C, overexpression of srRNA-4674-19 significantly increased the luciferase activity of PPARγ promoter reporter. ATP is a very important metabolic product and previous research showed hepatic ATP was downregulated in patients with type 2 diabetes [39]. We then detected the effect of srRNAs on intracellular ATP level. As shown in Figure 6D, srRNA-11714-25 significantly decreased the intracellular ATP level. All these data suggest that srRNAs are involved in the regulation of metabolic processes.

To detect whether srRNAs are also involved in other signaling pathways, we transfected srRNAs with luciferase reporter under the control of p53-responsive PUMA promoter into NIH/3T3 cells. As shown in Figure 6E, inhibition of srRNA-4672-21, srRNA-4674-19, srRNA-6901-18, srRNA-7007-19, srRNA-11095-26, srRNA-11958-25 and srRNA-11963-20 markedly increased the luciferase activity of PUMA promoter, suggesting these srRNAs are involved in p53 signaling pathway or other pathways involved in PUMA transcriptional activation. ERK pathway plays an important role in the transmission of cellular proliferation and developmental signals [40], and then we detected the effect of srRNAs on ERK pathway including the phosphorylation of ERK1/2, p90RSK, Elk-1 and p70S6K. As shown in Figure 6F, overexpression of srRNA-11714-25 increased the phosphorylation levels of ERK1/2, p90RSK, Elk-1 and p70S6K. These data suggest that srRNAs participate in a broad range of biological processes.

## Discussion

In this study, we systematically investigated and characterized srRNAs detected in some small RNA high-throughput sequencing datasets, and provide new insights for the potential functions of srRNAs in biological and pathological processes.

With the robust high-throughput sequencing technology, many small RNA expression profiles have been revealed, and available from some public database, such as GEO [41]. Using some small RNA expression datasets from GEO, we identified and characterized srRNAs, which were usually neglected [15], [16], [17], [26]. Actually, some known small RNAs, such as piRNAs including mouse piR-16, piR-38, piR-165, piR-170 and piR-171, are mapped to rDNA, and we found that a total of 60 mouse piRNAs are also srRNAs (Table S13). Moreover, some miRNAs including mouse miR-696, miR-712, miR-714 and miR-715 are also mapped to rDNA, and we found that a total of 10 mouse miRNAs are also srRNAs (Table S14). In addition, as shown in Table S15, 62 srRNAs are perfectly matched to piRNAs, including piR-16, piR-38, piR-170 and piR-171. Interestingly, some small RNAs, such as piR-38, piR-171, miR-696, miR-712 and miR-714 observed in the datasets we analyzed, can only be mapped to rDNA but not the reference genome. The overlap of srRNA with piRNAs and miRNAs demonstrate that at least some srRNAs are not generated from random degradation of rRNAs. Compared with other small RNAs, such as miRNA and piRNA, srRNAs coding from the same start position in rDNA usually have various length, which forms an interesting character of srRNA. srRNAs are mainly distributed in rDNA regions coding rRNAs (Figure 1C, 1D, 3, 5E and S2), and the different density of srRNAs in rDNA coding regions provide further evidence that a large number of srRNAs are not generated from random degradation of rRNA. We can’t exclude the possibility that some of the detected srRNAs in total small RNA might be generated in the small RNA isolation procedure, in which some rRNAs are degraded. However, our results demonstrate that a large number of srRNAs should be generated endogenously, at least for the srRNAs identified from AGO protein complexes in different species.

Although we systematically identified and characterized srRNAs, how srRNAs are generated is still largely unknown. It has been suggested that srRNAs can be generated as diRNA in DNA double-strand break repair [14]. Since srRNAs are mainly distributed in rDNA regions coding rRNAs (Figure 1C, 1D, 3, 5E and S2), it is unlikely that srRNAs are mainly generated as diRNA or derived from pre-rRNA degradation. It has been reported that rRNA was highly degraded in a chloramphenicol- and rifampin-dependant manner when *Salmonella* strains enters stationary phase [42]. Moreover, in *Escherichia coli* cells, rRNA degradation increases dramatically under conditions leading to slow cell growth, and rRNA degradation also happens in growing cells to eliminate rRNA molecules with defects in length, processing, folding or assembly [43], [44]. These previous reports show that rRNA can be endogenously degraded, suggesting that srRNA might be generated from endogenous degradation of rRNA. It has been proposed that rRNA is fragmented by endoribonucleases, and the resulting fragments are further degraded to mononucleotides by exoribonucleases for recycling [45], [46]. Our findings suggest that degradation of rRNA may also produce functional srRNAs except recycling mononucleotides. It has been reported that the exoribonucleases RNase II and RNase R are important for rRNA degradation during starvation in *Escherichia coli* cells, whereas RNase R and PNPase are more important for rRNA degradation in quality control [45]. These findings suggest that these RNases might be involved in the generation of different srRNAs under different conditions. The binding of srRNA with AGO proteins shown in Figure 3 implicates that some known small RNA processing pathway might be involved in the generation of srRNAs. Various sizes of rRNA fragments usually can be detected in previous report [47], and it is very difficult to distinguish whether some of them are endogenous transcripts or degraded fragments. Nevertheless, at least the srRNAs identified from AGO protein complexes should be mainly endogenous products. In addition, in mouse and human reference genome, there are a lot of rDNA fragments, whether these rDNA fragments can be transcribed is still largely known. Thus, we still can’t completely exclude the possibility that some srRNAs might be generated from transcripts different from full-length rRNA precursor as their specific precursors like miRNA [48]. All these interesting possibilities need to be studied in the future.

Different classes of small RNAs including miRNAs and piRNAs have various important biological functions [49], [50]. Similarly, in this study, we demonstrate that some srRNAs are correlated with diabetes and involved in various biological processes including glucose and energy metabolism (Figure 5 and 6). Interestingly, srRNAs in the normal mouse liver had a high peak at the beginning of 28S rRNA coding region, while srRNAs in diabetic mouse liver had a high peak at the end of 28S rRNA coding region (Figure 5E). However, the underlying mechanism for such a big change of the srRNAs in diabetic mouse liver is still unclear. miRNA expression profile has been proposed as diagnostic marker for disease [51], [52]. Our observation of the correlation between srRNA expression profile and diabetes suggests that srRNA expression profile should also be beneficial for disease diagnosis. Although we show that some srRNAs have biological functions by overexpression of their mimics or antisense oligonucleotides, the underlying mechanism is still largely unknown. Whether some srRNAs function like siRNA in post-transcriptional regulation or like miRNA in regulation of mRNA stability and translation [8], is yet to be elucidated in the future.

In summary, we initiated a systematic analysis of srRNAs, revealed a global view of srRNAs in multiple samples including some srRNAs in AGO protein complexes, and provided new evidences for the potential functions of srRNAs in physiological and pathological processes.

## Materials and Methods

### Dataset collection

In this study, we used 6 Solexa small RNA sequencing datasets obtained from GEO (Gene Expression Omnibus, http://www.ncbi.nlm.nih.gov/geo/index.cgi), including GSM304914, GPL7059, GSM531974, GSM531975, GSM531976 and GSM533911 [53], [54], [55]. This study also used 2 Solexa small RNA sequencing datasets of normal (GSM1054168) and diabetic (GSM1054169) mouse liver from our lab. Additionally, we collected a few high-throughput sequencing datasets containing small RNAs co-immunoprecipitated with AGO proteins from GEO, including GSM889256, GSM889279, GSM642335, GSM642337, GSM280087, GSM280088, GSM850202, GSM850203 and GSM850204 [14], [31], [32], [33]. GSM889279 contains two replicates, which were referred as GSM889279a and GSM889279b in this study. The brief information about the samples was provided in Table S16.

### Animal experiments

All animal experimental procedures were approved by the Institutional Animal Care and Use Committee of the Institute for Nutritional Sciences (Protocol number 2010-AN-9). 12 male C57BL/6 mice at the age of 7 weeks were purchased from SLAC (Shanghai, China), and 12 diabetic male db/db mice at the age of 7 weeks were purchased from Model Animal Research Center of Nanjing University (Nanjing, China). All the mice were allowed to have access to water and diets *ad libitum*. About 3 h after the beginning of light cycle, 9-week-old mice were sacrificed, and the livers were immediately removed and snap-frozen in liquid nitrogen.

### Solexa sequencing

For Solexa sequencing, total RNA was extracted from liver samples with Trizol reagent (Invitrogen), according to the manufacturer’s instruction. The quality and integrity of the total RNA was evaluated by electrophoresis on 1.2% agarose gel and Agilent 2100 BioAnalyzer (Agilent). The Solexa sequencing procedure was conducted by using the manufacturer’s protocol. Briefly, after gel purification of small RNAs sized at 18–30 nt, a pair of Solexa proprietary adaptors was ligated to their 5′ and 3′ ends. Ligation products were gel-purified, reverse transcribed, and amplified by PCR using Illumina’s small RNA primer set complementary to the linker sequences. The generated cDNA library was utilized for sequencing analysis using the Illumina 1G Genome Analyzer according to the manufacturer’s instructions.

### Bioinformatics analysis of small RNA datasets

For each of the datasets mentioned above, we filtered the Solexa small RNA sequencing dataset by eliminating low quality reads and trimming 3′ primer adaptor sequences and adaptor contaminants to generate clean reads using Cutadapt 1.0 [56]. Then we collected the clean reads sized at 18–30 nt for further analysis. The clean reads were aligned to the mouse reference genome (NCBI v37, mm9) or the human reference genome (NCBI v37, hg19) using Bowtie 0.12.5 [57] with the following options: –f –v 0 –a. The indicated clean reads were aligned to human ribosomal DNA repeat unit (GenBank: U13369) [28] and mouse rDNA repeat unit (GenBank: BK000964) [29] using Bowtie with the following options: –f –v 0 –m 1. The clean reads that could be perfectly aligned were considered as mapped reads, and the remaining were considered as unmapped reads. Considering a high density of simple sequence repeats and transposable elements of rDNA [29], some reads (averagely about 0.53% in total reads mapped to rDNA) mapped to multiple positions in rDNA unit were removed by Bowtie. The count of each unique srRNA was normalized to transcripts per million (TPM) in the clean reads mapped to corresponding reference rDNA. In this study, unique reads refer to different types of reads, and redundant reads refer to total reads.

The abundance distribution of srRNAs in rDNA unit was analyzed by F-seq 1.0 [58]. The top 20 abundantly expressed srRNAs were selected according to their average abundance in the two indicated samples. To compare the srRNA expression level in normal and diabetic samples, a total of 64723 srRNAs were used to calculate fold changes and false discovery rate (FDR) [59]. The srRNAs with an average abundance no less than 10 TPM between two samples, a fold change no less than 1.5 and FDR less than 0.01 were considered to be differentially expressed.

As several homogeneous rDNA gene fragments are contained in current genome assemblies of *Arabidopsis thaliana* and *Drosophila melanogaster*, thus all of the clean reads were directly aligned to the *Arabidopsis* ribosomal DNA repeat unit (GenBank: X52322) and *Drosophila* rDNA repeat unit (GenBank: M21017) to analyze srRNAs.

To investigate srRNAs co-immunoprecipitated with AGO proteins, the count of each unique srRNA was normalized to TPM in the total detected reads, and the distribution of srRNAs in rDNA unit was visualized by SigmaPlot 11.0 software.

### Plasmids

The PEPCK and G6Pase luciferase reporters driven by PEPCK and G6Pase promoter respectively were generous gifts from Dr. Yong Liu (Institute for Nutritional Sciences, CAS, Shanghai, China). To construct the luciferase reporter driven by PPARγ promoter as described previously [60], PPARγ promoter was amplified from mouse genomic DNA using the primers 5′-AGCCCGGGCTGCAGGAATTCGATGGATAGCAGTAACATTTTG-3′ and 5′- CCAAGCTTGATCAGCATAAAACAGAGATTTG-3′. Then the amplified DNA fragment was cloned into the SmaI/HinDIII sites of pGL3-basic vector (Promega). The PUMA luciferase reporter was kindly provided by Dr. Wei Gu (Columbia University) [61]. pSV40-β-gal was obtained from Promega.

### srRNA mimics and srRNA antisense oligonucleotides

A total of 10 and 13 srRNAs mainly with big fold change and abundance change distributed at different regions of rDNA unit were selected to synthesize srRNA mimics and antisense oligonucleotides respectively. srRNA mimics and 2′-O-methylated single-stranded srRNA antisense oligonucleotides (srRNA inhibitors) were obtained from GenePharma (Shanghai, China). To equalize the total amount of small RNA, irrelevant miRNA (cel-miR-239b) or its 2′-O-methylated single-stranded antisense oligonucleotide was co-transfected and indicated as negative control.

### Cell culture and transfection

Hepa 1–6 and NIH/3T3 cells obtained from ATCC were grown in DMEM with 25 mM glucose and 10% fetal bovine serum. For transfection, Hepa 1–6 or NIH/3T3 cells were transfected with the indicated srRNA mimics or srRNA inhibitors at the final concentration of 50 nM using Lipofectamine 2000 (Invitrogen) according to the manufacturer’s instruction.

### Luciferase assay

Hepa 1–6 or NIH/3T3 cells in 24-well plates were co-transfected with the indicated srRNA mimics or inhibitors (50 nM), luciferase reporter plasmid (0.1 µg/well) and pSV40-β-gal (0.1 µg/well) using Lipofectamine 2000. After transfection for 72 h, luciferase activities were determined by luciferase assay kit from Promega. Luciferase activity was normalized to β-galactosidase activity, which was determined as described previously [62].

### RNA isolation and analysis

Total RNA was extracted from the indicated samples using Trizol reagent. Equal amount of total RNA from each sample was analyzed by agarose gel electrophoresis with ethidium bromide staining.

### Measurement of ATP content

ATP levels were measured using an ATP Bioluminescent Assay Kit (Promega) and were normalized to the protein content.

### Western blot

Protein samples were analyzed using antibodies against phosphorylated Erk1/2, phosphorylated p90 RSK, phosphorylated Elk-1, phosphorylated p70 S6K (Cell Signaling), α-tubulin (Sigma) and secondary antibodies conjugated to horseradish peroxidase from Jackson ImmunoResearch. The bound immune complexes were detected with SuperSignal west pico chemiluminescent substrate (Pierce).

### Statistical analysis

Data are expressed as mean ± SD of at least three independent experiments. Statistical significance was assessed by Student’s t-test except indicated. Differences were considered statistically significant at p < 0.05.

## Supporting Information

Figure S1 **Venn diagrams summarizing the reads and percentage of small RNAs mapped to reference genome and rDNA unit in total reads.**
(TIF)

Figure S2 **A continuous tag sequence density estimation by F-Seq showed that srRNAs from mouse fetal liver (GSM533911) (A), human liver C (GSM531974) (B) and fibroblast (GSM850202) (C) were also mainly enriched in the regions coding 18S, 5.8S and 28S rRNA.**
(TIF)

Figure S3 **The comparison of srRNA length distribution in immunoprecipitated AGO protein complex and total small RNA.** (A) The length distribution of *Arabidopsis* seedling srRNAs and srRNAs co-immunoprecipitated with AGO2. (B) The length distribution of human fibroblast srRNAs, human fibroblast srRNAs co-immunoprecipitated with AGO proteins, and human liver srRNAs.
(TIF)

Figure S4 **The srRNA inhibitors had no significant effect on mature rRNA levels.** Total RNA was extracted from Hepa 1-6 cells transfected with the indicated srRNA inhibitors and their control, and analyzed by agarose gel electrophoresis and ethidium bromide staining. Anti-4674-21 and Anti-7007-19 match with 18S and 28S rRNA respectively. NC, negative control.
(TIF)

Table S1 **Mouse neutrophil srRNAs identified from Solexa small RNA sequencing dataset GSM304914.**
(XLS)

Table S2 **Mouse srRNAs identified from Solexa small RNA sequencing dataset GPL7059.**
(XLS)

Table S3 **Human liver srRNAs identified from Solexa small RNA sequencing dataset GSM531975.**
(XLS)

Table S4 **Human liver srRNAs identified from Solexa small RNA sequencing dataset GSM531976.**
(XLS)

Table S5 **The top 20 abundant mouse srRNAs in Solexa small RNA sequencing datasets GSM304914 and GPL7059.**
(XLS)

Table S6 **The top 20 abundant human liver srRNAs in Solexa small RNA sequencing datasets GSM531975 and GSM531976.**
(XLS)

Table S7 **The reads and percentage of total and unique srRNA in AGO protein complex and the controls.**
(XLS)

Table S8 **Human fibroblast srRNAs co-immunoprecipitated with AGO proteins.**
(XLS)

Table S9 **srRNAs identified in normal mouse liver.**
(XLS)

Table S10 **srRNAs identified in diabetic mouse liver.**
(XLS)

Table S11 **The top 20 abundant srRNAs in normal and diabetic mouse liver.**
(XLS)

Table S12 **Differentially expressed srRNAs in normal and diabetic mouse liver.**
(XLS)

Table S13 **piRNAs perfectly matched to mouse 45-kb rDNA unit.**
(XLS)

Table S14 **miRNAs perfectly matched to mouse 45-kb rDNA unit.**
(XLS)

Table S15 **Some mouse and human srRNAs are perfectly matched to piRNA.**
(XLS)

Table S16 **Brief information on the high-throughput sequencing datasets.**
(XLS)
