# Supplementary figures and images for: Profiling and Identification of Small rDNA-Derived RNAs and Their Potential Biological Functions
Source: PLoS One. 2013 Feb 13;8(2):e56842. doi: 10.1371/journal.pone.0056842 (PMC3572043; doi:10.1371/journal.pone.0056842)

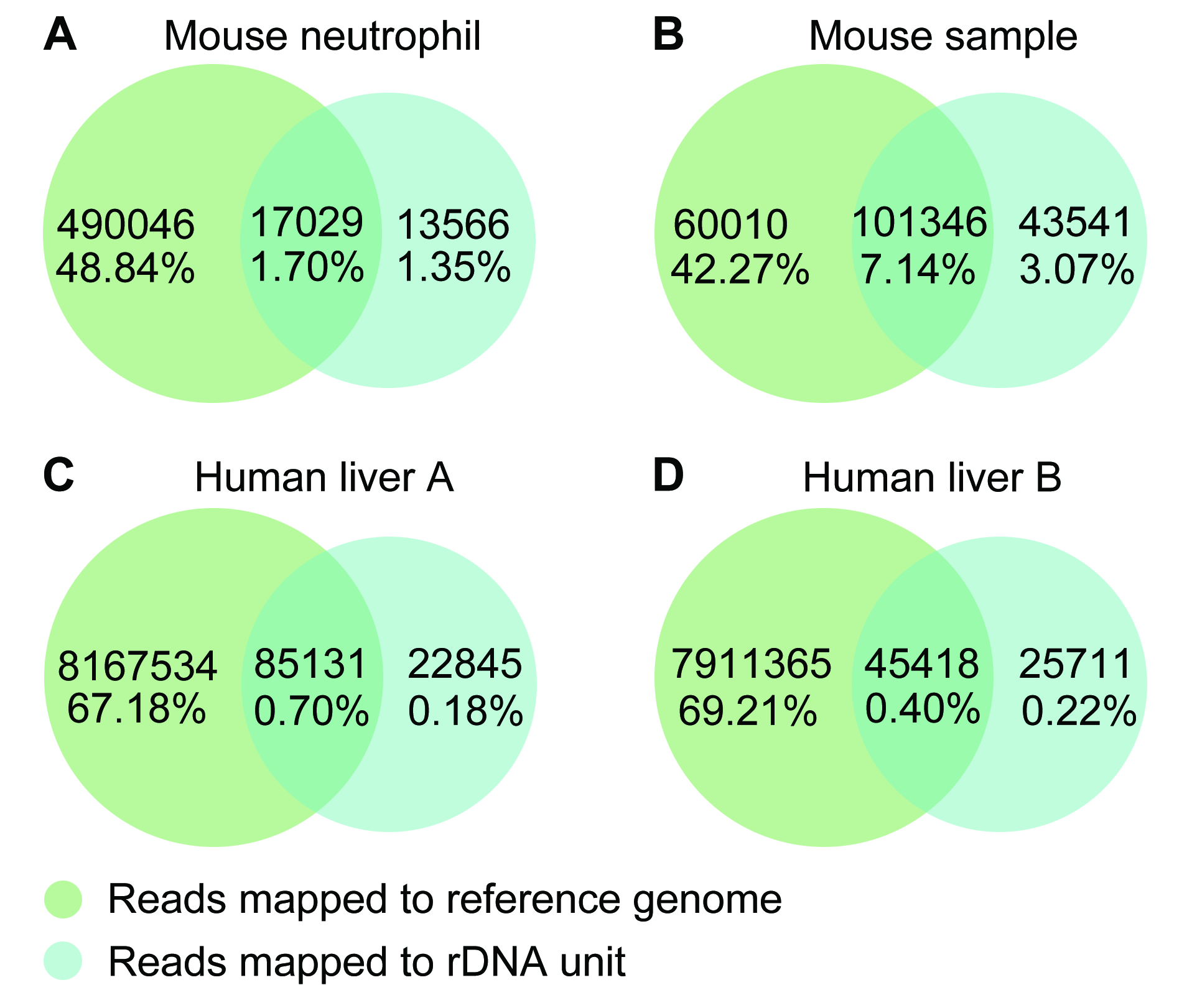

Supplement: Figure S1 — Venn diagrams summarizing the reads and percentage of small RNAs mapped to reference genome and rDNA unit in total reads. (TIF) [file pone.0056842.s001.tif]

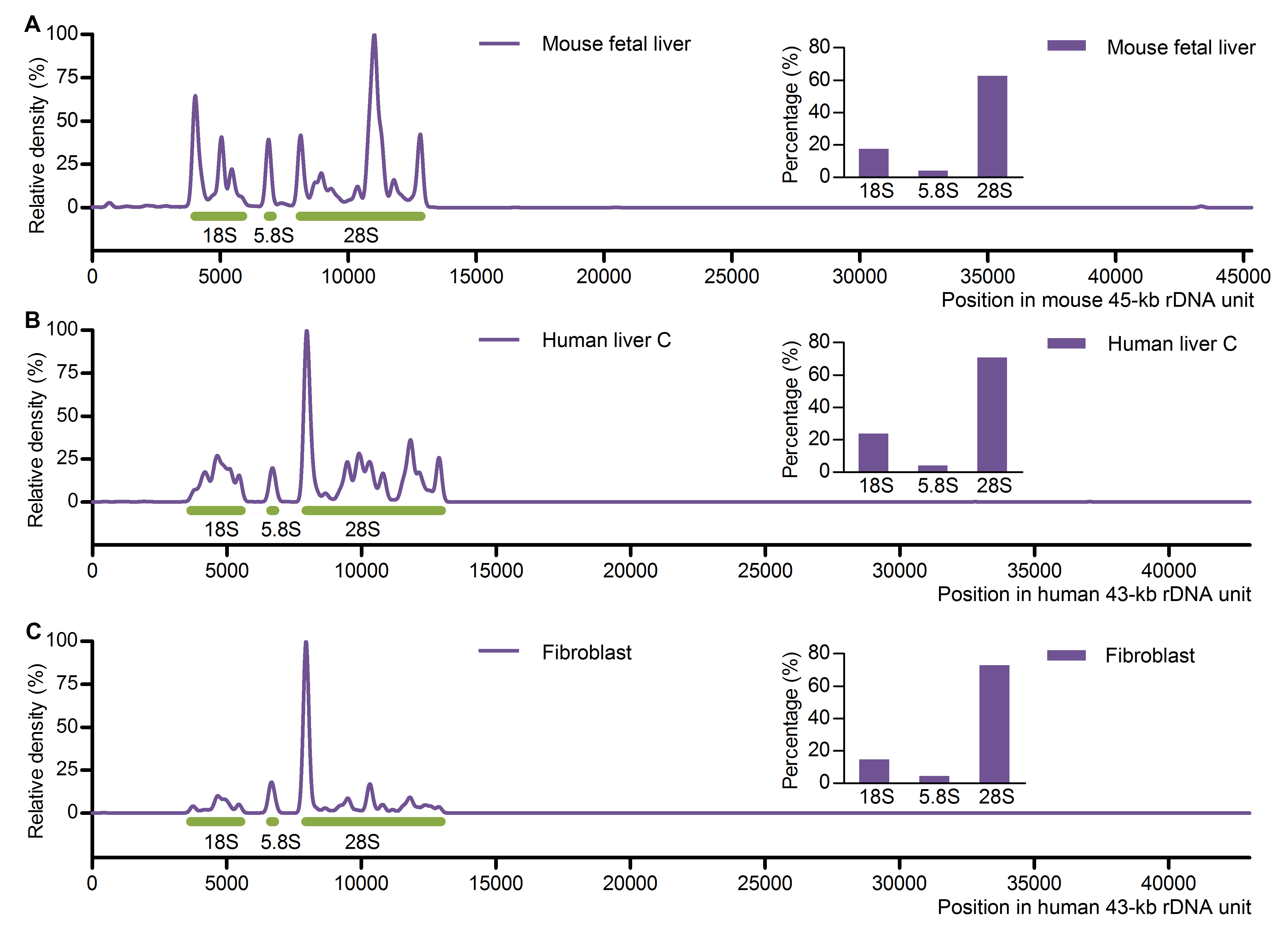

Supplement: Figure S2 — A continuous tag sequence density estimation by F-Seq showed that srRNAs from mouse fetal liver (GSM533911) (A), human liver C (GSM531974) (B) and fibroblast (GSM850202) (C) were also mainly enriched in the regions coding 18S, 5.8S and 28S rRNA. (TIF) [file pone.0056842.s002.tif]

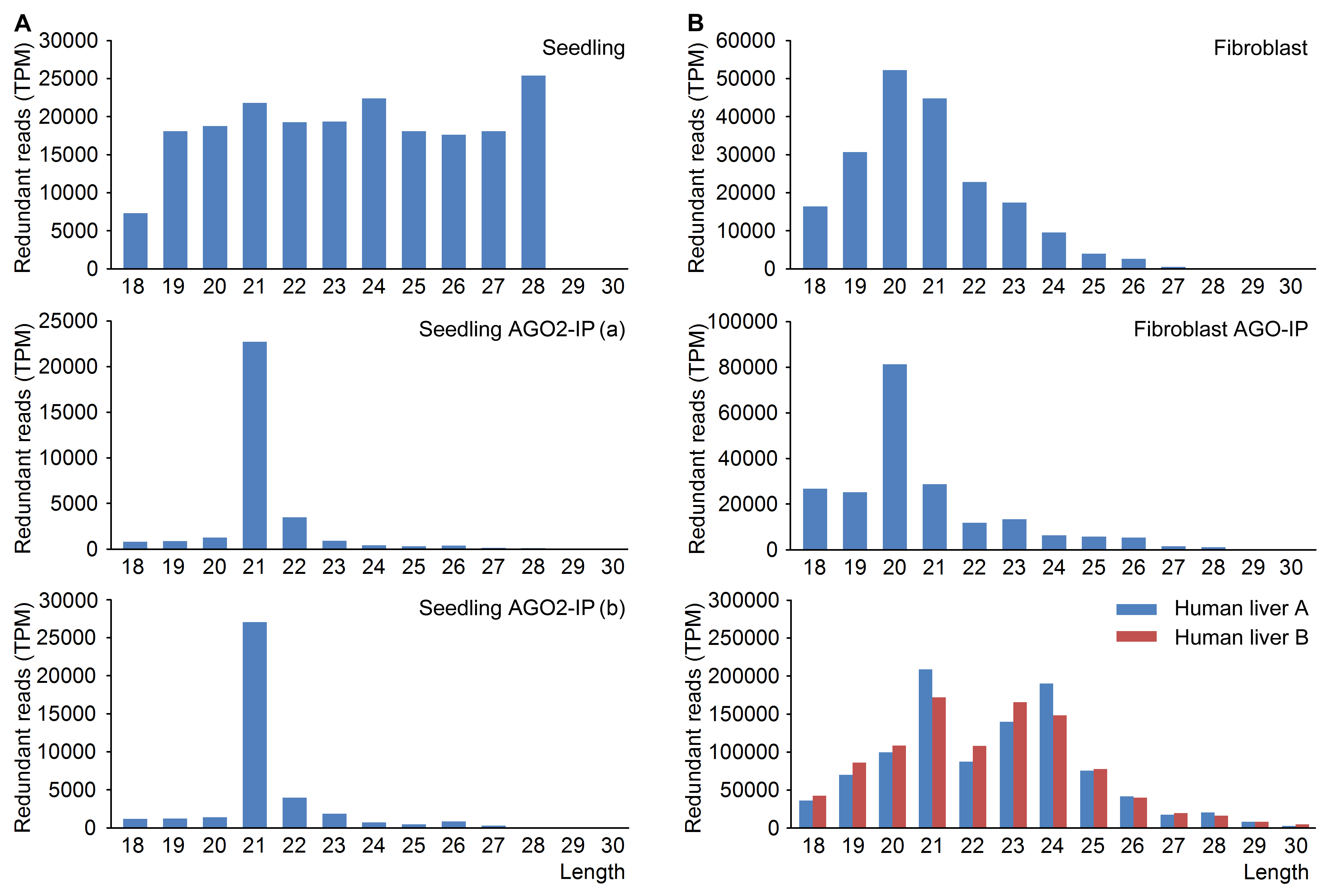

Supplement: Figure S3 — The comparison of srRNA length distribution in immunoprecipitated AGO protein complex and total small RNA. (A) The length distribution of Arabidopsis seedling srRNAs and srRNAs co-immunoprecipitated with AGO2. (B) The length distribution of human fibroblast srRNAs, human fibroblast srRNAs co-immunoprecipitated with AGO proteins, and human liver srRNAs. (TIF) [file pone.0056842.s003.tif]

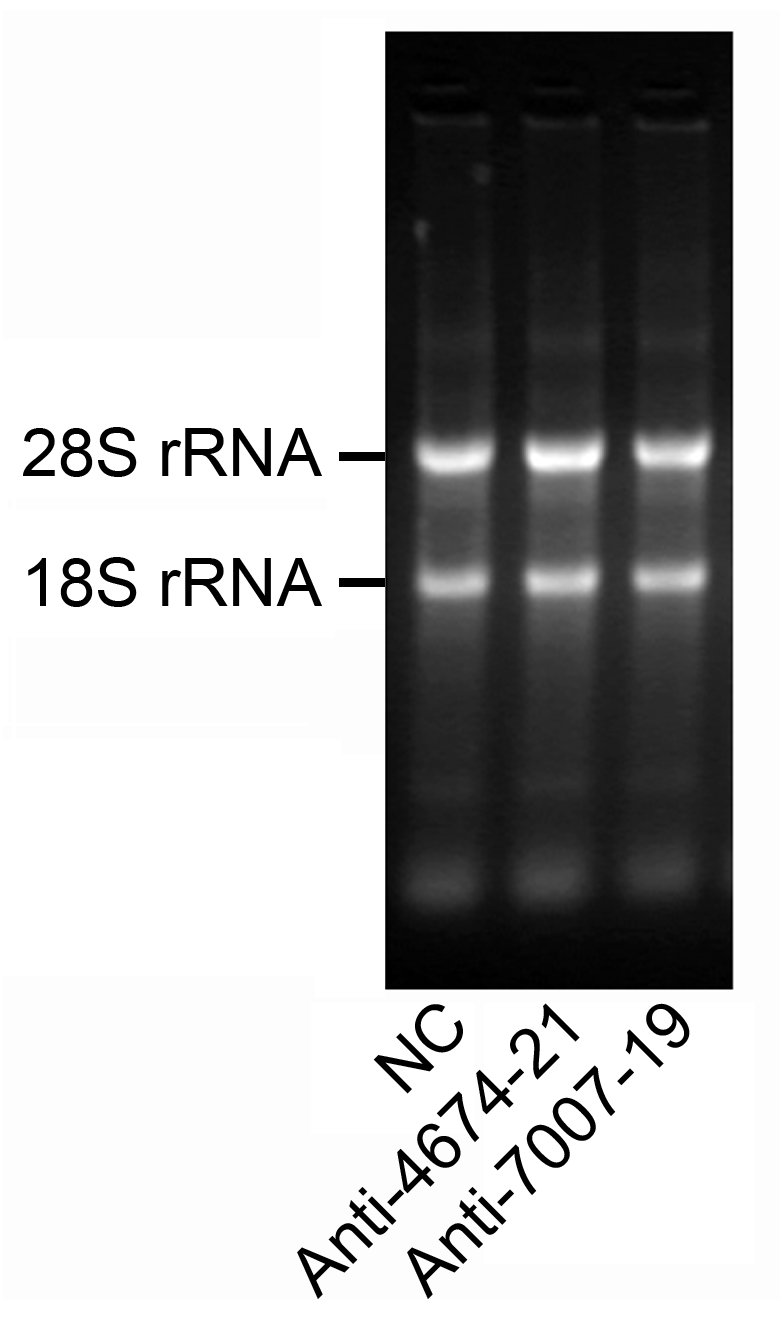

Supplement: Figure S4 — The srRNA inhibitors had no significant effect on mature rRNA levels. Total RNA was extracted from Hepa 1-6 cells transfected with the indicated srRNA inhibitors and their control, and analyzed by agarose gel electrophoresis and ethidium bromide staining. Anti-4674-21 and Anti-7007-19 match with 18S and 28S rRNA respectively. NC, negative control. (TIF) [file pone.0056842.s004.tif]
